# On the Employment of Iodide of Potassium in Diseases of the Brain in Children

**Published:** 1860-06

**Authors:** John Coldstream

**Affiliations:** M. D., F. R. C. P., Edin.


*On the employment of Iodide of Potassium in Diseases of the Brain in Children.* By JOHN COLDSTREAM, M. D., F. R. C. P., Edin.—It is now upwards of twenty years since iodide of potassium was commended by Rœser and others, as a remedy of special power in hydrocephalus. It is surprising how few seem to recognize its value, and what slight references are made to its employment in the various works on the diseases of children. In all cases when, from the course of the symptoms, there is reason to believe that the central organs of the nervous system, or their envelopes, are in any degree affected with strumous inflammation, (tubercular cerebritis, or meningitis,) or its consequences, after moderate purgation, the writer is in the habit of employing the iodide of potassium in doses of from half a grain to three grains, every three or four hours, in some carminative water, and continuing it for many days, according to the symptoms, or until convalescence is fully established; and with the occasioual use of blisters to the shaven scalp, he believes he has produced more prompt and decided effect upon the disease than by any other treatment. When the opportunity has been afforded of commencing the use of this remedy early, it has appeared to arrest the progress of the disease rapidly, so that the effects of effusion, indicated by squinting and convulsions, have not supervened. In less favorable circumstances, where considerable prostration has succeeded great febrile action, where starting and squinting had become prominent symptoms, in not a few instances, the free use of the iodide of potassium has been followed by amendment and complete recovery. In such cases it should be given in larger doses, even to four grains several times a day, to children of from four to eight years ot age.

The medicine is very seldom refused by the patient, nor does it increase the nausea so frequently existing in the earlier stages of the disease; nor has it induced salivation, which seems sometimes to follow its use in other ailments. Although ti is more especially useful where there exists more or less of the scrofulus diathesis, yet it has been found of service in cases where no taint was present.

The writer is not prepared to assert that this agent is more useful than calomel in *all* cases of inflammation of the brain and its appendages. When we have to treat robust and full-blooded children, in whom there is reason to believe that the threatened disease of the nervous system stands more or less directly connected with preceding disorder of the digestive organs, there is no doubt of the superior efficacy of the mercurial treatment, combined with antimonials and salines; but when, after having duly administered these remedies, symptoms of cerebral disorder continue, the iodide should then be employed. The writer, in concluding, is satisfied that the iodide of potassium never produces any bad effects, though it may fail to do good.—*Edinburgh Med. Jour.*, Dec., 1859.

